# Delivery workers’ subordinate work in delivery platform companies and street accidents: magnitude and associated factors

**DOI:** 10.1590/0102-311XEN204723

**Published:** 2025-03-24

**Authors:** Janaína Santos de Siqueira, Guilherme Loureiro Werneck, Paulo Gilvane Lopes Pena, Rita de Cássia Pereira Fernandes

**Affiliations:** 1 Instituto de Estudos em Saúde Coletiva, Universidade Federal do Rio de Janeiro, Rio de Janeiro, Brasil.; 2 Faculdade de Medicina da Bahia, Universidade Federal da Bahia, Salvador, Brasil.; 3 Instituto de Medicina Social, Universidade do Estado do Rio de Janeiro, Rio de Janeiro, Brasil.; 4 Instituto de Saúde Coletiva, Universidade Federal da Bahia, Salvador, Brasil.

**Keywords:** Occupational Accidents, Occupational Risks, Workplace Environment, Occupational Health, Motorcyclists, Accidentes de Trabajo, Riesgos Laborales, Ambiente de Trabajo, Salud de los Trabajadores, Motociclistas

## Abstract

This study investigated the magnitude of occupational accidents and associated factors among delivery workers working in Brazil via a web survey. The link to the questionnaire was disseminated via social media and in person. The prevalence of occupational accidents was estimated and Cox regression for cross-sectional studies provided crude and adjusted prevalence ratios to investigate the association between sociodemographic and occupational characteristics and occupational accidents. A total of 563 delivery workers took part in the web survey. A significant prevalence of occupational accidents in the last year was found, corresponding to 44.1%, which rose to 54.6% among those aged up to 28, 50% among those who work seven days per week and 49.1% among those who work more than 10 hours per day. Those who had experienced conflict with clients of delivery companies had a 51.3% prevalence of occupational accidents. This high magnitude, not found in studies of other professions, highlights the accident rate imposed by contemporary precarious work in delivery platform companies, carried out on the streets, given that 82.8% of these incidents were traffic accidents. Among other work factors, conflicts with company clients, lack of support from delivery platform companies and a fast pace were significantly associated with occupational accidents. The high magnitude, the association with the characteristics of the job, and the nature of the events on the streets are outcomes that call for intersectoral action to prevent them, addressing precarious work in delivery platform companies in order to promote decent work.

## Resumen

La magnitud de los accidentes laborales y los factores asociados entre los repartidores que trabajan en el territorio nacional se investigaron a partir de una encuesta en la cual se difundió el cuestionario mediante las redes sociales y presencialmente. Se estimaron las prevalencias de accidentes laborales; y la regresión de Cox en estudios transversales proporcionó razones de prevalencia brutas y ajustadas para investigar la asociación entre las características sociodemográficas, ocupacionales y de accidentes laborales. En la encuesta participaron 563 repartidores. Se encontró una prevalencia relevante de accidentes laborales en el último año aproximada del 44,1%, que llegó al 54,6% en las personas con menos de 28 años, el 50% entre las que trabajan como máximo siete días/semana y el 49,1% entre las que trabajan más de 10 horas/día. Aquellas personas que vivían en una situación de conflicto con los clientes de las empresas-plataformas de entregas tuvieron una prevalencia de accidentes laborales del 51,3%. Esta alta magnitud no se encuentra en estudios con otras categorías profesionales y muestra las precarias condiciones de accidentes laborales a que, actualmente, están expuestos los trabajadores de empresas-plataformas de reparto, realizado en las calles, dado que el 82,8% de estos incidentes fueron accidentes de tránsito. Entre otros factores de trabajo, los conflictos con los clientes de la empresa, la falta de apoyo de las empresas de plataformas de entrega y el ritmo rápido se asociaron con los accidentes laborales de manera relevante. La alta magnitud, la asociación con las características del trabajo y la naturaleza de los eventos en el espacio callejero son resultados de este estudio y requieren una acción intersectorial para su prevención desde un enfoque en el trabajo precario en empresas de reparto con el fin de promover el trabajo digno.

## Introduction

Delivery workers in delivery platform companies are a vulnerable category, given the organization of work in the Platform Economy, carried out on the streets, with two-wheeled vehicles, motorized or not ^1^ ^,^ ^2^. Evidence indicates that precarious work favors the occurrence of occupational accidents ^3^ ^,^ ^4^. Moreover, the new organizational arrangements at work, while reproducing old forms of worker subordination, imply patterns of occupational exposure that should be investigated as to their association with occupational accidents ^1^ ^,^ ^3^ ^,^ ^4^ ^,^ ^5^.

Occupational accidents are a public health problem with high morbidity and mortality, and their frequent underreporting tends to worsen among informal workers ^6^. Studies on occupational accidents among delivery workers are still lacking, despite the rapid growth of this category, especially in delivery platform companies. From 2016 to 2021, the estimated growth in the number of motorcyclists delivering goods in the country is 1,072% ^7^. Considering self-employed delivery workers, Lapa ^8^ estimated 678,527 delivery workers in the first year of the pandemic and a recent Brazilian Institute of Geography and Statistics (IBGE, acronym in Portuguese) study ^9^ estimated 589,000 workers on digital delivery platforms in 2022.

Although the precarious conditions of informal work for motorcycle couriers had already been noted prior to the consolidation of delivery platform companies, their rapid growth can now be observed ^1^. Some characteristics of delivery work indicate the expansion of occupational accidents in this category, as this work lacking regulation does not ensure the minimum conditions of protection during the working day ^1^. Workers with less experience and training opportunities, most of them with insufficient and unstable income, working on demand with payment per delivery and low remuneration, find a scenario of increased social unprotection, which includes the weakening of regulatory mechanisms related to health and safety at work ^5^ ^,^ ^10^.

An analysis of remuneration, representation, employment relationship, management and working conditions in Brazil have shed light to the unfair working conditions in platform companies, including delivery platform companies ^11^. In addition, there have been challenges in making decent work compatible with the organization of work in delivery platform companies ^12^.

Investigating the magnitude of occupational accidents among delivery workers is a necessary agenda, given the scarce knowledge about this category, which so far has meant dealing with evidence of the problem mainly through studies that focus on traffic accidents involving motorcyclists in general, disregarding their working status. Knowing the factors associated with occupational accidents and their repercussions among delivery riders might help develop surveillance strategies for workers’ health and support interventions focused on prevention. These actions are in line with the global agenda for sustainable development, which includes decent work as one of its goals ^12^.

This study aims to investigate the magnitude of occupational accidents and the factors associated with these events among delivery workers.

## Methods

### Procedures and study population

The *EpisSAT Entregadores* web survey was carried out in accordance with the procedures recommended for this type of survey ^13^ ^,^ ^14^. Data collection began in February 2022 and lasted four months.

The study population was made up of workers who deliver goods, without restriction to a type of employment relationship, across Brazil. In view of the unavailability of data with any precision on the population employed as delivery workers in Brazil, as well as the lack of a common record of this population accessible to researchers, this study considered reaching at least a non probabilistic sample, adopting strategies that would enable access to a population that is difficult to reach ^15^, which would also make a census of the category unfeasible. These are methodological innovations, given the precarious working conditions of delivery workers, which also discourages them from answering questionnaires ^15^.

Participation was voluntary, with no material or financial incentives. Representants of delivery workers were approached in all regions of Brazil from the planning stage of the survey. Such approach made it possible, among other things, to adapt the language and format of the questionnaire with the collaboration of some of these individuals, in order to minimize potential information bias.

The link to access the questionnaire was shared on social media - WhatsApp, Telegram, Instagram, and Facebook. The web survey was also publicized in person, with the questionnaire link being sent to WhatsApp and provided in person by delivery riders at street gatherings, mainly in Salvador, Bahia State, Brazil. Conducting online and in person dissemination was a relevant action to reinforce adherence, in order to have repercussions on the response of the category in general.

The online questionnaire in this study investigated sociodemographic, occupational and occupational accidents characteristics.

### Occupational accident outcome

Understood in its broadest sense ^6^, occupational accident was defined in the questionnaire as any situation during work as a delivery worker (including commuting to work and returning home) that has caused an injury, hurt, trauma, or health problem.

Participants answered the question: How many accidents at work have you suffered in the last 12 months, i.e., in the last year? If they ticked “none”, they were directed to the next section of the questionnaire. Participants who ticked any of the other alternatives, ranging from one to five or more accidents, were considered to have had an occupational accident in the last year. The occupational accident were characterized regarding magnitude; circumstance (traffic accident, interpersonal violence, or other forms of trauma); leave of absence and its length; use of health services and their nature, and the support received after the event. Participants were asked to consider the most serious occupational accident if they had suffered more than one occupational accident in the last year.

### Covariables

The following sociodemographic variables were investigated: age, sex, race/skin color, schooling, state, and income. The following occupational variables were included: length of time working; whether working as a delivery person is the main activity; whether it is the only paid activity; number of days worked in the last week; average daily working hours in the last week; number of companies in which they work as a delivery person; employment relationship; the vehicle used; social security contributions; helmet use; running red lights in traffic, and psychosocial demands - support from the company and support from colleagues to solve problems at work, fast pace, conflicts with suppliers of goods and conflicts with company clients.

### Statistical analyses of data

After data collection, duplicate responses were checked by comparing the internet protocol number of the respondent’s device.

In the descriptive analysis, data completeness was verified, with minimal losses in some variables and a higher proportion of losses for the variables “employment relationship” (platform company, freelance/private, cooperative, formal contract) and “age”, which was subjected to multiple imputation. In the tables with the descriptive results, the number (n) of each variable was recorded, in order to clarify any losses. The means and dispersion measures and medians of the quantitative variables were obtained, as well as the absolute and relative frequencies of occupational accidents, according to sociodemographic and occupational variables.

The prevalence of occupational accident in the previous year was then estimated. Occupational accident is a non-insidious event, often estimated in cross-sectional studies using the incidence measure ^16^. However, in view of the length of the time frame used (one year) in which it is possible to accumulate new and old cases for the same individual, the frequency of the condition was estimated based on its prevalence.

In the analytical analysis, multiple imputation with chained equations was carried out due to the loss of 16.95% (n = 93) of answers for “age” ^17^. It was considered that the loss of answers was not due to missing data, and the assumption of randomness of the missing data was accepted, which allows for use of imputation. The automatic method was used, with a simple linear regression with 10 iterations, defining the same limits found for the variable in the original database. There were no significant differences in the magnitudes of the associations in the models after multiple imputation.

The variables were categorized as follows: age into three strata delimited according to tertiles; race/skin color, initially as white, black, brown, Indigenous and yellow and, for the analytical stage, recategorized as black and non-black, taking into account the prevalence of work accidents in the strata; education as complete high school or more and until complete high school; working time as a delivery worker, which was investigated in three categories in the descriptive stage, up to 3 years, from 4 to 7 years and over 7 years, and recategorized, in the analytical stage, into up to 7 years and over 7 years; weekly working days, categorized in the descriptive stage into up to 5 days, 6 days and 7 days and, in the analytical stage, recategorized into up to 6 days and 7 days of work in the last week; and daily working hours of up to 8 hours, over 8 to 10 hours and over 10 hours. The psychosocial characteristics of work, regarding the use of helmets and running red lights in traffic were measured as frequently, rarely and never. After checking the distribution of the original strata, the variables support from the company, support from colleagues and fast pace at work were dichotomized as frequently and rarely/never. The remaining variables were stratified as yes (often/rarely) and no (never).

Cox regression for cross-sectional studies provided crude and adjusted prevalence ratios (PR). In high-prevalence binary outcomes, the odds ratio provided by logistic regression tends to be overestimated. In this context, Cox regression for cross-sectional studies is easy to perform and yields valid estimates of PR as indicated in the literature ^18^ ^,^ ^19^.

The investigation of factors associated with accidents was carried out using multiple analysis based on six models according to repercussions and number of occurrences in the last year, also with the aim of identifying factors associated with accidents severity, which may suggest new lines of investigation and intervention in this emerging problem. In these models, individuals were compared: those who had been injured once in the last year with those who had not; those who had been injured twice or more in the same period with those who had not; injured individuals who were off work with those who were not injured, and injured individuals who were off work with those who were not injured. Finally, accident victims who reported having used healthcare services as a result of the occupational accident were compared with non-injury victims.

The independent variables were included in the models according to biological and epidemiological plausibility criteria, based on occupational literature, in three blocks: sociodemographic, occupational, and psychosocial.

Procedures compatible with the non-probabilistic nature of the study population were adopted. Thus, results interpretation primarily considered the frequency and association measures obtained, although the 95% confidence interval (95%CI) is available in the tables, not interpreted as a dichotomous indicator of statistical significance. Furthermore, given the convenience sample, the results of the multiple analysis should be cautiously analyzed, provided that it is not possible to exclude the possibility of some bias in the results ^20^ ^,^ ^21^ ^,^ ^22^ ^,^ ^23^.

The data was analyzed using IBM SPSS, version 21.0 (https://www.ibm.com/).

### Ethical aspects

The study was approved by the Research Ethics Committee (Protocol n. 5.258.142), following the Brazilian National Health Council *Resolution n. 466/2012* for research involving human beings.

## Results

A total of 563 delivery workers took part in the study. They were predominantly men (96.3%), young, with half of them aged under 33 and almost 80% under 40.

Approximately 70% of the delivery workers were mixed-race (47.3%) or black (23%), and had completed high school. There were participants from all regions of Brazil, covering 21 states and the Federal District, with the Northeast (46.2%) and Southeast (36.5%) regions predominating (Table 1).

**Table 1:** Prevalence of occupational accidents according to sociodemographic characteristics among delivery workers. *EpisSAT Entregadores*, Brazil, 2022 (N = 563).

Variables	Total population		Prevalence of at least one occupational accident in the last year	
	n	%	n	%
Sex [n = 560]				
Male	542	96.8	238	43.9
Female	18	3.2	10	55.6
Age (years) [n = 563]				
15 to 28	184	32.7	100	54.6
> 28 to 37	200	35.5	85	41.9
> 37	179	31.8	63	34.8
Race/Skin color [n = 562]				
Black	129	23.0	67	51.9
Mixed-race	266	47.3	112	42.1
White	145	25.8	60	41.4
Yellow	15	2.7	5	33.3
Indigenous	7	1.2	3	42.9
Schooling [n = 562]				
Until complete high school	172	30.6	74	43.0
Complete high school or more	390	69.4	174	44.6
Region of Brazil in which you work [n = 561]				
Central-West	40	7.1	24	60.0
Northeast	259	46.2	119	45.9
North	11	2.0	4	36.4
Southeast	205	36.5	77	37.6
South	46	8.2	23	50.0

Most participants stated wearing helmet (88%) while working, but 67.6% admit to running red lights in traffic. During their daily journey, more than 83% of the workers do not have the support of the delivery platform company to solve problems they face at work. Moreover, 72% reported conflicts with company clients and 70.5% experienced conflicts with suppliers of goods (Table 2).

**Table 2:** Prevalence of occupational accidents according to occupational characteristics among delivery workers. *EpisSAT Entregadores*, Brazil, 2022 (N = 563).

Variables	Total population		Prevalence of at least one occupational accident in the last year		
	n	%	n	%
Employment as a delivery worker [n = 449]				
Digital platform	391	87.1	165	42.2
Other employment realtionship	58	12.9	25	43.1
Main activity [n = 559]				
Yes	503	90.0	227	45.1
No	56	10.0	18	32.1
Only activity [n = 558]				
Yes	435	78	197	45.3
No	123	22	49	39.8
Working time as a delivery worker (years) [n = 550]				
Up to 3	303	53.8	143	47.2
From 4 to 7	120	21.3	59	49.9
Over 7 years	140	24.9	46	32.9
Weekly working days [n = 553]				
Up to 5	168	30.4	67	39.9
6	149	26.9	60	40.3
7	236	42.7	118	50.0
Daily working hours [n = 508] *				
Up to 8	148	29.1	59	39.9
From 8 to 10	134	26.4	59	44.0
Over 10	226	44.5	111	49.1
Vehicle used [n = 563] **				
Bicycle	65	11.6	26	40.0
Motorcycle	495	88.4	222	44.8
Characteristics of the vehicle used [n = 561]				
Own	531	94.7	233	43.9
Rented	23	4.1	10	43.5
Others	7	1.2	4	57.1
Storage of goods [n = 562]				
Bag	331	58.9	168	50.8
Fixed trunk	212	37.7	77	36.3
Others	19	3.3	3	15.8
Social Security [n = 556]				
Yes	280	50.4	103	36.8
No	276	49.6	143	51.8
Helmet use [n = 523]				
Yes	460	88.0	210	45.7
No	63	12.0	26	41.3
Running red lights [n = 552]				
Yes	373	67.6	191	51.2
No	179	32.4	54	30.2
Company support [n = 561]				
Often	93	16.6	31	33.3
Rarely/Never	468	83.4	216	46.2
Support from colleagues [n = 556]				
Often	282	50.6	129	45.7
Rarely/Never	275	49.4	117	42.5
Fast pace [n = 558]				
Often	389	69.7	190	48.8
Rarely/Never	169	30.3	57	33.7
Conflicts with suppliers [n = 556]				
Yes	392	70.5	191	48.7
No	164	29.5	56	34.1
Conflict with company clients [n = 550]				
Yes	396	72.0	203	51.3
No	154	28.0	43	27.9
Total population	563	100.0	248	44.1

* There were 13 missing cases and we excluded 4 individuals who had not worked in the last week and 38 who worked more than 15 hours a day;

** Three individuals who used another type of vehicle were excluded.

Younger delivery workers, those who work seven days per week, those who run red lights and those who report conflicts with customers had the highest prevalence of at least one occupational accident, equal to or greater than 50%, and this increased as the daily working day increased (Tables 1 and 2).

Delivery workers carry out deliveries as their main paid activity (90%) or as their only paid activity (78%), mainly in delivery platform companies (87.1%). Around 70% of delivery workers work six or seven days per week, with daily working hours of 10 hours or more for 50% of the participants (Table 2). Among these, 37.2% worked 12 hours or more in the last week (data not shown). A significant proportion of the workers joined the category up to three years ago (53.8%), which corresponds mainly to the pandemic period. Almost all of them make deliveries by motorcycle (88.4%). Around half do not contribute to Social Security.

There was a 44.1% prevalence of at least one occupational accident in the last year among delivery workers. Traffic accidents accounted for 82.8% of these accidents. The body regions most affected were legs (27.7%), arms (21.1%), hands and fingers (19.4%) and feet (16.5%). More than half of the injured workers (56.1%) reported taking time off work due to an occupational accident, and among those who were off work for 15 days or more (22.2%), only 20% received social security benefits. Regardless of the length of time off work, help from family members to cover expenses was the most frequently reported (38.3%). The health services used because of the occupational accident were almost always public (84.8%), urgent and emergency (92.6%) (data not shown).

From the results of the multiple analysis, some findings stand out due to the remarkable magnitude of the association.

Experiencing conflicts with delivery platform companies clients was associated with almost double the occurrence of accidents without time off work as compared to those who denied such conflicts (PR = 1.96). Having delivery work as their main paid activity and not having the support of the company to resolve problems arising from work increased the occurrence of accidents without time off by 60% and almost 50%, respectively. Another association with this outcome was black race/skin color (PR = 1.42) (Table 3).

**Table 3:** Factors associated with occupational accidents according to the need for time off work in the last year. *EpisSAT Entregadores*, Brazil, 2022 (N = 563).

Variables	Occupational accident without time off work versus without occupational accident [n = 423]					Occupational accident with time off work versus without occupational accident [n = 453]				
	Prevalence (%)	Crude PR	95%CI	Ajusted PR	95%CI	Prevalence (%)	Crude PR	95%CI	Adjusted PR	95%CI
Age (years)										
> 37	20.0	1.00	-	1.00	-	22.1	1.00	-	1.00	-
> 28 to 37	27.2	1.35	0.83-2.21	1.29	0.77-2.19	26.5	1.20	0.74-1.94	1.10	0.65-1.87
15 to 28	30.0	1.50	0.90-2.48	1.14	0.64-2.04	43.1	1.95	1.26-3.03	1.61	0.95-2.73
Race/Skin color										
Non-black	23.6	1.00	-	1.00	-	28.3	1.00	-	1.00	-
Black	32.6	1.38	0.91-2.11	1.42	0.90-2.25	37.4	1.32	0.90-1.92	1.21	0.80-1.84
Main activity										
No	15.6	1.00	-	1.00	-	22.4	1.00	-	1.00	-
Yes	26.6	1.71	0.79-3.68	1.60	0.63-4.06	31.2	1.39	0.75-2.57	1.38	0.69-2.79
Working time as a delivery worker (years)										
Over 7	18.3	1.00	-	1.00	-	20.3	1.00	-	1.00	-
Up to 7	28.2	1.55	0.96-2.49	1.36	0.77-2.42	34.0	1.67	1.08-2.60	1.17	0.68-2.02
Weekly working hours										
Up to 6	23.7	1.00	-	1.00	-	26.1	1.00	-	1.00	-
7	29.3	1.24	0.85-1.81	1.05	0.68-1.61	36.6	1.40	1.00-1.96	1.14	0.78-1.67
Daily working hours										
Up to 8	20.5	1.00	-	1.00	-	28.2	1.00	-	1.00	-
From 8 to 10	24.2	1.18	0.67-2.09	1.05	0.57-1.93	31.8	1.13	0.71-1.80	1.15	0.71-1.88
Over 10	31.1	1.52	0.93-2.48	1.16	0.67-2.01	33.5	1.19	0.78-1.81	0.92	0.58-1.46
Running red lights										
No	18.3	1.00	-	1.00	-	17.2	1.00	-	1.00	-
Yes	30.3	1.65	1.07-2.54	1.37	0.85-2.21	37.7	2.19	1.43-3.35	1.78	1.11-2.84
Company support										
Often	16.2	1.00	-	1.00	-	23.5	1.00	-	1.00	-
Rarely/Never	27.4	1.69	0.93-3.08	1.49	0.78-2.85	32.1	1.37	0.84-2.22	1.28	0.75-2.19
Fast pace										
Rarely/Never	20.0	1.00	-	1.00	-	20.6	1.00	-	1.00	-
Often	28.7	1.43	0.93-2.20	1.05	0.65-1.71	35.2	1.71	1.13-2.58	1.36	0.84-2.21
Conflicts with company clients										
No	12.6	1.00	-	1.00	-	19.7	1.00	-	1.00	-
Yes	32.3	2.56	1.51-4.36	1.96	1.12-3.45	36.1	1.84	1.21-2.81	1.29	0.81-2.02

95%CI: 95% confidence interval; PR: prevalence ratio.

Red-light running represented an increase of almost 80% in the prevalence of occupational accident with time off work, which was also associated with age, with delivery workers aged up to 28 years showing a 60% higher prevalence than older peers (Table 3).

Making deliveries as a main activity and for up to 7 years represented an increase of almost 80% and 50%, respectively, in occupational accidents that did not lead to accessing healthcare services. Running red lights was also associated with the occurrence of occupational accidents that required seeking healthcare services (PR = 1.75) or not (PR = 1.41). Notably, younger delivery workers were more likely to suffer more serious occupational accidents, since they required individuals to seek healthcare (PR = 1.65). While the experience of conflicts with clients was associated with accupational accidents that did or did not lead to seeking healthcare services (PR = 1.46 and PR = 1.66, respectively), the lack of support from the company was mainly associated with occupational accidents that led to seeking care (PR = 1.45) (Table 4).

**Table 4:** Factors associated with occupational accidents according to the use of health services in the last year. *EpisSAT Entregadores*, Brazil, 2022.

Variables	Occupational accident without use of health service versus without occupational accident [n = 438]					Occupational accident with use of health service versus without occupational accident [n = 440]				
	Prevalence (%)	Crude PR	95%CI	Ajusted PR	95%CI	Prevalence (%)	Crude PR	95%CI	Adjusted PR	95%CI
Age (years)										
> 37	23.4	1.00	-	1.00	-	20.0	1.00	-	1.00	-
> 28 to 37	25.3	1.12	0.69-1.84	1.03	0.62-1.71	28.6	1.40	0.86-2.27	1.41	0.82-2.42
15 to 28	37.3	1.67	1.06-2.62	1.23	0.73-2.06	37.3	1.86	1.17-2.97	1.65	0.93-2.91
Race/Skin color										
Non-black	26.2	1.00	-	1.00	-	26.2	1.00	-	1.00	-
Black	34.7	1.32	0.89-1.97	1.24	0.79-1.95	35.4	1.35	0.91-2.00	0.74	0.48-1.13
Main activity										
No	17.4	1.00	-	1.00	-	20.8	1.00	-	1.00	-
Yes	29.0	1.67	0.81-3.42	1.79	0.71-4.45	29.2	1.40	0.73-2.68	1.23	0.61-2.50
Working time as a delivery worker (years)										
Over 7	19.0	1.00	-	1.00	-	20.3	1.00	-	1.00	-
Up to 7	31.4	1.65	1.04-2.62	1.50	0.85-2.64	31.4	1.54	0.99-2.61	0.99	0.57-1.49
Weekly working hours										
Up to 6	26.6	1.00	-	1.00	-	23.4	1.00	-	1.00	-
7	31.0	1.16	0.81-1.66	0.93	0.62-1.40	35.5	1.52	1.07-2.16	1.33	0.89-1.99
Daily working hours										
Up to 8	23.3	1.00	-	1.00	-	26.4	1.00	-	1.00	-
From 8 to 10	30.6	1.31	0.79-2.18	1.29	0.75-2.22	25.7	0.97	0.58-1.63	0.92	0.54-1.57
Over 10	31.5	1.35	0.85-2.15	1.11	0.67-1.86	33.5	1.27	0.82-1.95	0.93	0.58-1.49
Running red lights										
No	18.8	1.00	-	1.00	-	16.7	1.00	-	1.00	-
Yes	33.8	1.80	1.18-2.72	1.41	0.89-2.23	35.0	2.10	1.35-3.26	1.75	1.08 -2.84
Company support										
Often	20.5	1.00	-	1.00	-	19.5	1.00	-	1.00	-
Rarely/Never	29.6	1.44	0.85-2.44	1.25	0.70-2.23	30.4	1.56	0.91-2.67	1.45	0.80-2.61
Fast pace										
Rarely/Never	18.8	1.00	-		-	21.7	1.00	-	1.00	-
Often	32.8	1.74	1.13-2.68	1.28	0.78-2.08	31.8	1.47	0.98-2.21	1.16	0.72-1.86
Conflicts with company clients										
No	22.9	1.00	-	1.00	-	18.2	1.00	-	1.00	-
Yes	31.2	2.17	1.36-3.48	1.66	0.99-2.76	33.2	2.08	1.31-3.29	1.46	0.90-2.35

95%CI: 95% confidence interval; PR: prevalence ratio.

Running red lights and making deliveries as a main activity increased the prevalence of an occupational accident in the last year by approximately 60%, but were also associated with the occurrence of two or more occupational accidents in the last year (PR = 1.53 and PR = 1.41 respectively). Experiencing two or more occupational accidents in the last year was also more likely among those who work without company support (PR = 1.66), with a fast pace (PR = 1.56), and who are younger (PR = 1.44). Once again, the experience of conflict with company clients was associated with the occurrence of multiple occupational accidents in the period, almost doubling the prevalence as compared to delivery workers who had not experienced this stressful situation (PR = 1.90) (Table 5).

**Table 5:** Factors associated with one or two or more occupational accidents in the last year. *EpisSAT Entregadores*, Brazil, 2022.

Variables	One occupational accident in the last year versus without occupational accident [n = 419]					Two or more occupational accidents in the last year versus without occupational accident [n = 458]				
	Prevalence (%)	Crude PR	95%CI	Ajusted PR	95%CI	Prevalence (%)	Crude PR	95%CI	Adjusted PR	95%CI
Age (years)										
> 37	21.0	1.00	-	1.00	-	22.1	1.00	-	1.00	-
> 28 to 37	25.2	1.26	0.75-2.11	1.19	0.69-2.07	27.5	1.25	0.79-2.00	1.20	0.73-1.98
15 to 28	29.1	1.48	0.89-2.47	1.32	0.72-2.42	44.4	1.92	1.25-2.95	1.44	0.88-2.37
Race/Skin color										
Non-black	23.8	1.00	-	1.00	-	28.3	1.00	-	1.00	-
Black	28.7	1.21	0.77-1.89	1.34	0.82-2.18	40.4	1.43	0.99-2.04	1.27	0.85-1.89
Main activity										
No	15.6	1.00	-	1.00	-	22.4	1.00	-	1.00	-
Yes	26.0	1.67	0.78-3.6	1.58	0.63-3.99	32.0	1.43	0.77-2.64	1.41	0.70-2.85
Working time as a delivery worker (years)										
Over 7	20.3	1.00	-	1.00	-	19.0	1.00	-	1.00	-
Up to 7	26.6	1.31	0.83-2.06	1.22	0.69-2.15	35.4	1.86	1.18-2.94	1.28	0.74-2.21
Weekly working hours										
Up to 6	24.9	1.00	-	1.00	-	25.2	1.00	-	1.00	-
7	24.8	1.00	0.7-1.49	0.85	0.54-1.33	39.8	1.58	1.13-2.20	1.33	0.92-1.94
Daily working hours										
Up to 8	19.8	1.00	-	1.00	-	29.4	1.00	-	1.00	-
From 8 to 10	27.9	1.41	0.81-2.45	1.39	0.77-2.50	28.6	0.97	0.6-1.57	0.88	0.53-1.44
Over 10	27.2	1.37	0.82-2.29	1.22	0.70-2.12	36.8	1.25	0.84-1.87	0.86	0.55-1.35
Running red lights										
No	17.2	1.00	-	1.00	-	18.3	1.00	-	1.00	-
Yes	30.0	1.74	1.18-2.72	1.61	0.99-2.62	38.1	2.08	1.38-3.15	1.53	0.97-2.41
Company support										
Often	21.5	1.00	-	1.00	-	17.3	1.00	-	1.00	-
Rarely/Never	25.4	1.18	0.7-1.99	1.13	0.64-1.98	34.0	1.96	1.11-3.47	1.66	0.90-3.06
Fast pace										
Rarely/Never	22.8	1.00		1.00	-	17.6	1.00	-	1.00	-
Often	26.3	1.15	0.76-1.75	0.97	0.60-1.56	37.2	2.11	1.36-3.27	1.56	0.94-2.58
Conflicts with company clients										
No	17.8	1.00	-	1.00	-	14.6	1.00	-	1.00	-
Yes	29.3	1.65	1.04-2.06	1.24	0.76-2.02	38.7	2.65	1.63-4.30	1.90	1.14-3.17

95%CI: 95% confidence interval; PR: prevalence ratio.

Thus, experience of conflict with company clients, working as a delivery rider as their main paid activity and running red lights were the variables most consistently associated with the occurrence of occupational accidents. Furthermore, being younger, of black race/skin color, working as a delivery rider for up to 7 years, working at a fast pace and not receiving support from the company to solve job-related problems were associated with occupational accidents, either as a minor or supposedly more serious event.

## Discussion

The study showed a high prevalence of occupational accidents, reaching 54.6% among those aged up to 28, 50% among those who work seven days per week and 49.1% among those who work more than 10 hours per day. Those who had experienced conflict with delivery platform companies clients had a 51.3% prevalence of occupational accidents. Such outcome, which has not been identified in studies on other professions, shows the high accident rate caused by precarious work in delivery platform companies, to which 87.1% of the delivery workers in this study are linked, and carried out on the streets, given that 82.8% of these incidents were traffic accidents, with significant recurrence.

Contrary to the companies’ claims that delivery workers would work flexible hours and in a complementary way to other main occupations, it turned out that they are the sole or main source of income for a large and growing workforce in Brazil ^24^ ^,^ ^25^. Considering the size of this workforce, a proper regulation of the working conditions of the category should be created ^7^ ^,^ ^9^.

This population working on the streets has become a particularly vulnerable group as victims of traffic accidents. Official data - despite its fragility in terms of characterizing the occupation of those involved - shows a high proportion of traffic accidents affecting motorcyclists, reaching 30% mortality, with a growing trend in middle- and low-income countries ^2^.

Studying the occurrence of occupational accidents requires knowledge of the working hours to which the studied population is subject. Our findings reveal intense occupational exposure under the adverse conditions of precarious street work, resulting in significant repercussions on the health and safety of delivery workers ^4^ ^,^ ^26^.

The daily working hours found in this study are very long and higher than those found in the literature for other professions. Notably, a study carried out at the first months of the COVID-19 pandemic with delivery workers found less extensive working hours at that time ^10^. Since then, in addition to the discussion of the strenuous working hours of this category, its growing trend has been considered a consequence of increased competition due to the increase of delivery workers, as a result of the economic and health crises, but also the smaller payment by the delivery platform companies, as they monopolized the delivery sector and intensified the delivery workers’ dependence on platforms ^5^ ^,^ ^10^.

Working in traffic, driving two-wheeled vehicles, is full of stressors generated by sharing public roads with four-wheeled vehicles, under constant threat of an accident, given the lack of segregated lanes for motorcyclists or a safe system that promotes riskless ride ^2^. These stressors are compounded by those encountered in customer service, when the delivery worker is exposed to all types of dissatisfaction from a wide and diverse contingent of customers ^27^. In this unfavorable context of working on the streets and in traffic, there is also a lack of support from delivery platform companies to solve the problems faced at work, when dealing with adversities related to the digital platform and, above all, customer relations.

The prevalence of occupational accidents found in this study was more than 10 times that of the general employed population in Brazil ^28^ and higher than that found in closed environments - as healthcare workers - whose frequency of occupational accidents with biological material in the last year corresponded to 3.4% ^16^. Among motorcycle taxi drivers, the frequency of occupational accidents ranged from 10.5% ^29^ to 26.8% ^30^. A study conducted with motorcycle couriers by Silva et al. ^31^, prior to the expansion of delivery platform companies in Brazil, found a frequency of at least one occupational accident in traffic, particularly, at 37.1%. The magnitude of these abovementioned findings, which is already high, is still lower than the one found in this study, but it reinforces the unsafe working conditions in traffic, especially on motorcycles.

Regarding occupational accidents severity, more than half of the injured delivery workers had to take time off work. Among those who were off work for 15 days or more, 80% were not entitled to a paid period by the Social Security system to recover from the accident, in a situation of social unprotection, in which survival becomes a challenge for these workers. Moreover, the fact that most healthcare services sought were public - urgent and emergency care, given the acute nature of the event - indicates an increase in demand and costs for the Brazilian Unified National Health System (SUS, acronym in Portuguese) due to the uberized working conditions and their impact on health.

The diversity of approaches to the severity of occupational accidents in the literature limits the possibilities of comparison ^31^ ^,^ ^32^. It is likely that the severity of occupational accidents, estimated in this study by referring to absence from work and/or use of healthcare services, is underestimated, considering the lack of social protection and the likely lower access to healthcare services among platformized workers ^33^. The lack of, or difficulty in accessing, compensation insurance from delivery platform companies, and scarce access to Social Security, may force delivery workers to avoid taking time off work, or do so for a shorter period than is recommended for health recovery, due to the need to earn an income to survive ^5^ ^,^ ^31^ ^,^ ^32^. Seeking healthcare services can also be discouraged by the need to keep working ^5^ ^,^ ^33^. Data from the *Brazilian National Health Survey* ^34^ enabled us to estimate that poor access to healthcare services is higher among men, people aged 18 to 24, black people, and people of low socioeconomic status, a profile similar to that of the delivery workers in this study.

Similar to our findings, running red lights in traffic has been reported in other studies with delivery workers and is related to the time pressure imposed by delivery platform companies. Digital platform technologies boost such pressure, by remunerating individuals based on completed deliveries and an evaluation system that makes the receipt of new delivery orders conditional, among other factors, which is directly related to the speed of the service provided ^5^ ^,^ ^26^ ^,^ ^32^ ^,^ ^33^. However, the fact that 88% of the delivery workers in this study wore helmets, a personal protective equipment, shows that it is the objective working conditions that can favor or hinder the adoption of safety measures by workers, i.e., the real conditions are the determinants of a more or less safe working system ^32^ ^,^ ^35^.

Under time pressure, running a red light may be the only alternative, while wearing a helmet, which is often kept on the head even when getting off the motorcycle to receive or deliver goods, is feasible even when working intensively.

Being younger was associated with occupational accidents with time off work, use of healthcare services and recurrence within one year. The association between younger workers and occupational accidents is in line with previous studies with delivery workers ^31^ ^,^ ^32^ ^,^ ^33^. These findings do not seem to be linked to youth per se, but rather to fewer training opportunities in precarious work ^33^ ^,^ ^35^ ^,^ ^36^.

In this sense, greater susceptibility to work intensification, higher speeds, less attention to traffic rules due to lack of training for professional activity with motorcycles and less experience at work are some explanations for the higher incidence in this age group ^3^ ^,^ ^33^ ^,^ ^35^. In Greece, delivery workers aged 18 to 24 had more than double the risk (120% more) of running red lights ^32^. In another survey of 1,310 delivery workers with insurance benefits granted after a motorcycle accident in Korea, it was found that although workers under the age of 20 had a relevant prevalence of occupational accidents, this was lower than among workers over 30, suggesting underreporting of events among younger delivery workers, with less access to insurance benefits and therefore underrepresented in the study population ^36^.

In this study, there was no difference in the frequency of occupational accidents between whites and browns, which is why they were analyzed together as non-blacks, as well as yellows and indigenous people, even though the disadvantages of blacks and browns in the world of work are recognized. Delivery workers of black race/skin color were more likely to be victims of accidents that occurred without leave.

Making deliveries as a main paid activity was mainly associated with occupational accidents of supposedly lesser severity and which did not recur over a period of one year. Dependence on this source of income can potentiate the intensification of work, culminating in long working hours, and has been associated with occupational accidents ^5^. The potentiation of the effects of work stressors in long working hours and the fatigue resulting from maintaining abnormal postures favor unsafe conditions when piloting ^26^ ^,^ ^33^ ^,^ ^37^ ^,^ ^38^.

Making deliveries for up to seven years was associated with occupational accidents without the use of health services. This period coincides with the expansion of delivery platform companies, suggesting that not only a shorter period of experience, but also joining the category in this context of increased precariousness may have compromised safety when working on two-wheeled vehicles ^5^ ^,^ ^7^ ^,^ ^36^ ^,^ ^37^.

Psychosocial stressors at work have been associated with occupational accidents, including its more serious repercussions. When present, social support makes it possible, to some extent, to protect the worker from the effects of the high psychological burden in conditions of low autonomy to manage their own work, as is the case with delivery workers ^39^. Among health workers, the experience of low support contributed to a 24% increase in the occurrence of accidents involving biological material, compared to high social support ^16^. It was associated as well with a higher occurrence of road traffic accidents in two out of three groups of professional drivers - city and intercity bus drivers ^40^.

The accelerated pace of work, which was associated with the recurrence of occupational accidents, is a consequence of the time requirements at work. The evaluation by the companies’ clients, one of the components of a score that conditions the service offered, includes punctuality in delivery based on travel times estimated by the companies ^33^ ^,^ ^41^. In addition, the high productivity required, with the application of gamified targets, stimulates the acceleration of the pace of work ^41^. This scenario favors the use of high speeds in motor vehicles, a recognized factor associated with traffic accidents and the adoption of behaviors related to the greater severity of occupational accidents ^26^ ^,^ ^32^. Chen ^37^ found that time pressure at work, as well as being the biggest single predictor of stress, increased the influence of work overload on this health problem. This, consequently, contributed to the development of behavior considered unsafe when driving in traffic, such as running red lights.

In this study, running red lights was associated with more serious accidents. Knowing the real conditions in which delivery riders carry out their tasks is necessary in order to avoid reducing the complexity of the findings with individual and behavioral explanations, without identifying the economic and social determinants of intensified work ^32^.

The street is an inhospitable work environment, in which workers are isolated, without the resources usually found in limited work environments, such as the factory or office, to deal with conflicting situations ^6^. Given the association found between psychosocial stressors and different outcomes related to occupational accidents, especially conflicts with clients, mechanisms to ensure support for delivery workers to solve work-related problems, considering the street space, should have some protective effect. This support could help reduce conflicts with customers.

There was a concentration of occupational accidents in traffic. Further epidemiological studies should investigate the different types of occupational accidents and the recognized underreporting of interpersonal violence as occupational accident on the street ^6^. Furthermore, it is possible that the fear of suffering violence contributes to running red lights in traffic, which was associated with occupational accident in this study.

Our results should contribute to workers’ health surveillance, in line with the global agenda for sustainable development, which includes among its objectives decent work and improving road safety and reducing traffic deaths ^12^ ^,^ ^42^.

### Strengths and limitations of the study

No previous studies have been identified that have investigated the magnitude and factors associated with occupational accidents and their repercussions, such as time off work and use of health services, in Brazil. Therefore, our evidence makes a contribution to the literature to characterize and elucidate the magnitude of occupational accidents among delivery workers, especially in platform work.

The strategies adopted to make the study possible are a strength, given the difficulties in accessing this population and the lack of willingness on the part of delivery platform companies to provide society with data on this workforce ^10^ ^,^ ^15^.

The methodological procedures adopted were aimed at minimizing potential information and selection bias. It is possible that delivery workers away from work due to occupational accident did not access the link to the questionnaire posted in delivery worker groups on WhatsApp and social media. However, the permanence and recurrence of this link on the category’s social media for four months may have minimized this possible selection bias, the healthy worker survival effect.

Given the impossibility of carrying out a census and the possible non-probabilistic sample selection strategy, it is impossible to rule out the possibility of differences between participating and non-participating delivery workers, requiring caution when generalizing the results. However, non-participation does not seem to have been the result of a refusal associated with a particular reason. On the contrary, it is precarious work that discourages them from dedicating time to answering the survey. In this sense, it is likely that those who had the opportunity to hear more about the project, in the face-to-face and online dissemination actions, felt more encouraged to take part. The restriction of face-to-face dissemination to two states was due to the lack of funding for the research from development agencies. In this sense, the results achieved, which highlight the seriousness of these workers’ health situation, compensate for the great effort made to make the research possible.
