# Supplementary material for: Delivery workers’ subordinate work in delivery platform companies and street accidents: magnitude and associated factors
Source: Cad Saude Publica. 2025 Mar 24;41(3):e00204723. doi: 10.1590/0102-311XEN204723 (PMC11940858; doi:10.1590/0102-311XEN204723)
Supplement: Translation

# Translation: Trabalho subordinado de entregadores em empresas-plataforma de entrega e acidentes nas ruas: magnitude e fatores associados

**DOI:** 10.1590/0102-311XPT204723

**Authors:** Janaína Santos de Siqueira, Guilherme Loureiro Werneck, Paulo Gilvane Lopes Pena, Rita de Cássia Pereira Fernandes

**Affiliations:** 1 Instituto de Estudos em Saúde Coletiva, Universidade Federal do Rio de Janeiro, Rio de Janeiro, Brasil.; 2 Faculdade de Medicina da Bahia, Universidade Federal da Bahia, Salvador, Brasil.; 3 Instituto de Medicina Social, Universidade do Estado do Rio de Janeiro, Rio de Janeiro, Brasil.; 4 Instituto de Saúde Coletiva, Universidade Federal da Bahia, Salvador, Brasil.

**Keywords:** Acidentes de Trabalho, Riscos Ocupacionais, Ambiente Externo de Trabalho, Saúde do Trabalhador, Motociclistas

## Abstract

Investigou-se a magnitude dos acidentes de trabalho e os fatores associados entre entregadores de mercadorias atuando no território nacional por meio de *websurvey*, cujo *link* para o questionário foi divulgado por mídias sociais e presencialmente. Estimaram-se as prevalências de acidentes de trabalho e a regressão de Cox para estudos de corte transversal forneceu razões de prevalência brutas e ajustadas para investigação da associação entre características sociodemográficas, ocupacionais e acidentes de trabalho. Participaram da *websurvey* 563 entregadores. Constatou-se relevante prevalência de acidentes de trabalho no último ano, correspondente a 44,1%, que chegou a 54,6% naqueles com até 28 anos de idade, 50% entre quem trabalha a jornada máxima de sete dias por semana e 49,1% entre quem trabalha mais de dez horas por dia. Aqueles que viveram situação de conflito com clientes das empresas-plataforma de entrega tiveram prevalência de acidentes de trabalho de 51,3%. Tal elevada magnitude, não encontrada em estudos com outras categorias profissionais, evidencia a acidentalidade imposta pelo trabalho precário contemporâneo em empresas-plataforma de entrega, executado nas ruas, haja vista que 82,8% dessas ocorrências foram acidentes de trânsito. Dentre outros fatores do trabalho, conflitos com clientes das empresas, escassez de apoio das empresas-plataforma de entrega e ritmo acelerado se associaram aos acidentes de trabalho de forma relevante. A alta magnitude, a associação com as características do trabalho e a natureza dos eventos no espaço das ruas são resultados deste estudo que exigem a intersetorialidade para sua prevenção, com abordagem do trabalho precário em empresas-plataforma de entrega, para promoção do trabalho decente.

## Introdução

Os entregadores de mercadorias em empresas-plataforma de entrega compõem uma categoria vulnerabilizada tendo em vista a organização do trabalho na Economia de Plataforma, desenvolvido no espaço das ruas e com veículos de duas rodas, motorizados ou não ^1^ ^,^ ^2^. Há evidências de que o trabalho precário favorece a ocorrência de acidentes de trabalho ^3^ ^,^ ^4^. Ademais, os novos arranjos organizacionais no trabalho, ainda que reproduzindo antigas formas de subordinação dos trabalhadores, implicam padrões de exposição ocupacional que devem ser investigados quanto à sua associação com acidentes de trabalho ^1^ ^,^ ^3^ ^,^ ^4^ ^,^ ^5^.

Os acidentes de trabalho são um problema de saúde pública de elevada morbimortalidade e sua frequente subnotificação tende a se agravar entre trabalhadores informais ^6^. Estudos sobre acidentes de trabalho com entregadores ainda são uma lacuna, a despeito do rápido crescimento da categoria, sobretudo em empresas-plataforma de entrega. De 2016 a 2021, estima-se um crescimento de 1.072% especificamente no número de motociclistas entregadores de mercadorias no país ^7^. Considerando os trabalhadores de entrega por conta própria, Lapa ^8^ estimou 678.527 trabalhadores de entrega no primeiro ano da pandemia e estudo recente do Instituto Brasileiro de Geografia e Estatística (IBGE) ^9^ estimou 589 mil trabalhadores em plataformas digitais de entrega em 2022.

Ainda que já fossem constatadas condições precárias da informalidade do trabalho de *motoboys* anteriores à expansão das empresas-plataforma de entrega, observa-se na contemporaneidade sua rápida ampliação ^1^. Algumas características do trabalho de entregadores indicam a ampliação dos acidentes de trabalho nessa categoria, pois esse trabalho desregulamentado não assegura as condições mínimas de proteção no curso da jornada ^1^. Trabalhadores com menor experiência e oportunidade de capacitação, em sua maioria, com renda insuficiente e instável, realizando um trabalho sob demanda com pagamento por entrega de baixa remuneração, encontram um cenário de desproteção social ampliada, com renda insuficiente e instável, que inclui fragilização dos mecanismos regulatórios relacionados à saúde e à segurança no trabalho ^5^ ^,^ ^10^.

Condições injustas no trabalho em empresas-plataforma, incluindo as empresas-plataforma de entrega, foram evidenciadas a partir da análise da remuneração, da representação, das formas de contratação, da gestão e das condições de trabalho no Brasil ^11^. Além disso, têm sido constatados desafios para compatibilizar o trabalho decente com o a organização do trabalho nas empresas-plataforma de entrega ^12^.

A investigação da magnitude dos acidentes de trabalho com entregadores é uma agenda necessária, haja vista a lacuna desse conhecimento sobre a categoria que impõe, até o momento, lidar com as evidências do problema principalmente por meio de estudos que abordam acidentes de trânsito com motociclistas em geral, sem identificar se estão ou não em situação de trabalho. Conhecer os fatores associados aos acidentes de trabalho e suas repercussões entre entregadores deve possibilitar desenvolver estratégias de vigilância em saúde do trabalhador e subsidiar intervenções com foco na prevenção. Essas ações estão alinhadas com a agenda global para o desenvolvimento sustentável, que tem o trabalho decente como um dos seus objetivos ^12^.

O presente estudo se propõe a investigar a magnitude dos acidentes de trabalho e os fatores associados a esses eventos entre entregadores de mercadorias.

## Métodos

### Procedimentos e população do estudo

Realizou-se uma *websurvey*, o *EpisSAT Entregadores*, conforme procedimentos preconizados para esse tipo de inquérito ^13^ ^,^ ^14^. A coleta de dados teve início em fevereiro de 2022, com duração de quatro meses.

A população do estudo foi composta por trabalhadores que realizam entrega de mercadorias, sem restrição a um tipo de relação de trabalho, no território nacional. Em vista da indisponibilidade de dados com alguma precisão relativos à população ocupada como entregadores de mercadoria no Brasil, bem como a inexistência de registro comum dessa população acessível aos pesquisadores, este estudo considerou atingir ao menos uma amostra de natureza não probabilística, adotando estratégias que viabilizassem o acesso a uma população de difícil alcance ^15^, o que também tornaria pouco factível um censo da categoria. Tais estratégias são inovações metodológicas diante da precarização do trabalho de entregadores, que também desestimula a resposta a questionários ^15^.

A participação dos entregadores foi voluntária, sem incentivos materiais ou financeiros. Buscou-se o diálogo com lideranças de entregadores desde a etapa de planejamento do inquérito em todas as regiões do Brasil. Esse procedimento possibilitou, dentre outros fatores, adequar a linguagem e o formato do questionário com a colaboração de algumas dessas lideranças, a fim de minimizar potenciais vieses de informação.

O *link* para acesso ao questionário foi divulgado em mídias sociais - WhatsApp, Telegram, Instagram e Facebook. Houve divulgação presencial da *websurvey* com envio do *link* do questionário ao WhatsApp fornecido presencialmente pelos entregadores em locais de concentração nas ruas, principalmente em Salvador, Bahia, Brasil. A condução de etapas híbridas de divulgação, *online* e presencial, foi uma ação relevante de reforço à adesão, a fim de repercutir na resposta da categoria em geral.

Do questionário *online* no presente estudo, foram investigadas as características sociodemográficas, ocupacionais e sobre acidentes de trabalho.

### O desfecho: acidente de trabalho

Compreendido em sua concepção ampliada ^6^, o acidente de trabalho foi definido no instrumento de pesquisa como qualquer situação durante o trabalho como entregador (incluindo o deslocamento para o trabalho e o retorno para casa) que tenha gerado alguma lesão, machucado, trauma ou problema de saúde.

Os participantes responderam à questão: Quantos acidentes de trabalho você sofreu nos últimos 12 meses, ou seja, de um ano para cá? Ao assinalarem “nenhum”, eram direcionados para a seção subsequente do questionário. Foram considerados caso de acidentes de trabalho no último ano os participantes que assinalaram qualquer uma das demais alternativas, variando de um a cinco ou mais acidentes de trabalho. Os acidentes de trabalho foram caracterizados quanto à magnitude, à circunstância (acidente de trânsito, violência interpessoal ou outras formas de trauma), ao afastamento e sua duração, à utilização de serviço de saúde e sua natureza e quanto ao apoio recebido após a ocorrência. Os participantes foram orientados a considerar o acidentes de trabalho mais grave caso tivessem sofrido mais de um no último ano.

### Covariáveis

Investigaram-se variáveis sociodemográficas (idade, sexo, raça/cor, escolaridade, Unidade da Federação e renda) e ocupacionais, como tempo de trabalho, se o trabalho como entregador é a principal atividade, se é a única atividade remunerada, quantidade de dias trabalhados na última semana, jornada diária média de trabalho na última semana, quantidade de empresas nas quais trabalha como entregador, vínculo como entregador, veículo utilizado, contribuição para a Previdência Social, utilização do capacete, ultrapassagem de sinal vermelho no trânsito e demandas psicossociais (apoio da empresa e apoio dos colegas para resolução de problemas no trabalho, ritmo acelerado, conflitos com fornecedores das mercadorias e conflitos com clientes das empresas).

### Abordagem estatística dos dados

Após obtenção dos dados, procedeu-se ao controle de duplicidade de respostas mediante comparação do número *Internet Protocol* (IP) do dispositivo do respondente.

Na etapa descritiva, verificou-se a completude dos dados, constatando-se perdas mínimas em algumas variáveis e maior proporção de perdas para as variáveis “modalidade de inserção no trabalho de entrega” (em empresa-plataforma, *freelancer*/particular, em cooperativa, com carteira assinada) e “idade”, que foi submetida à imputação múltipla. Nas tabelas com os resultados descritivos, registrou-se o quantitativo (n) de cada variável, a fim de elucidar a perda que tenha havido. Foram obtidas as médias e medidas de dispersão e as medianas das variáveis quantitativas, bem como as frequências absolutas e relativas de acidentes de trabalho, de acordo com variáveis sociodemográficas e ocupacionais.

Em seguida, estimou-se a prevalência de acidentes de trabalho no último ano. O acidente de trabalho é um evento não-insidioso, frequentemente estimado em estudos de corte transversal através da medida de incidência ^16^. No entanto, tendo em vista a extensão do recorte temporal utilizado (o período de um ano), no qual é possível acumular casos novos e antigos para o mesmo entregador, procedeu-se à estimativa da frequência do agravo considerando-a prevalência.

Na etapa analítica, imputação múltipla com equações encadeadas foi conduzida devido à perda de 16,95% (n = 93) de respostas para a variável “idade” ^17^. Considerou-se que a perda de respostas não ocorreu em função dos dados não observados, sendo aceita a suposição de aleatoriedade dos dados omissos, que permite o uso da imputação. Foi empregado o método automático, com regressão linear simples com dez iterações, definindo-se os mesmos limites encontrados para a variável no banco original. Não houve diferenças substanciais nas magnitudes das associações nos modelos após a imputação múltipla.

As variáveis foram categorizadas como se segue: idade em três estratos delimitados de acordo com os tercis; raça/cor da pele, inicialmente como brancos, pretos, pardos, indígenas e amarelos e, para etapa analítica, recategorizada em pretos e não pretos, tendo em vista a prevalência de acidentes de trabalho nos estratos; escolaridade como Ensino Médio completo ou mais e até Ensino Médio completo; tempo de trabalho como entregador, que foi investigada em três categorias na etapa descritiva, até 3 anos, de 4 a 7 anos e acima de 7 anos, e recategorizada, na etapa analítica, em até 7 anos e acima de 7 anos; jornada semanal, categorizada na etapa descritiva em até 5 dias, 6 dias e 7 dias e, na etapa analítica, recategorizada em até 6 dias e 7 dias de trabalho na última semana; e jornada diária em até 8 horas, acima de 8 até 10 horas e acima de 10 horas. As características psicossociais do trabalho, sobre o uso de capacete e ultrapassagem do sinal vermelho no trânsito foram mensuradas como frequentemente, raramente e nunca. Após verificação da distribuição dos estratos originais, foram dicotomizadas como frequentemente e raramente/nunca as variáveis apoio da empresa, apoio dos colegas e ritmo acelerado no trabalho. As demais variáveis foram estratificadas como sim (frequentemente/raramente) e não (nunca).

Regressão de Cox para estudos transversais forneceu razões de prevalência brutas e ajustadas (RP). Em desfechos binários de elevada prevalência, a razão de chance fornecida pela regressão logística tende a ser superestimada. Nesse contexto, a regressão de Cox para estudos de corte transversal é de fácil execução e provê estimativas válidas da RP conforme apontado na literatura ^18^ ^,^ ^19^.

A investigação dos fatores associados aos acidentes de trabalho foi conduzida na análise múltipla a partir de seis modelos conforme repercussões e quantidade de ocorrências no último ano, com o fim também de identificar fatores associados à gravidade do acidente de trabalho, o que pode sugerir novas linhas de investigação e de intervenção sobre esse problema emergente. Nesses modelos, compararam-se indivíduos: acidentados uma vez no último ano com não acidentados, acidentados duas ou mais vezes no mesmo período com não acidentados, acidentados que não se afastaram do trabalho com aqueles não acidentados, bem como os acidentados que se afastaram do trabalho com aqueles não acidentados. Por fim, compararam-se acidentados que referiram ter utilizado serviços de saúde em decorrência do acidente de trabalho com aqueles não acidentados.

As variáveis independentes foram inseridas nos modelos conforme critérios de plausibilidade biológica e epidemiológico, com base na literatura ocupacional, em três blocos: sociodemográficas, ocupacionais e psicossociais.

Adotaram-se procedimentos compatíveis com a natureza não probabilística da população de estudo. Assim, a interpretação dos resultados considerou primordialmente as medidas de frequência e de associação obtidas, embora estejam disponíveis nas tabelas os intervalos de 95% de confiança (IC95%), não interpretados como indicador dicotômico de significância estatística. Ademais, dada a amostra de conveniência, os resultados da análise múltipla devem ser analisados com a cautela exigida nesses casos, em que não é possível excluir a possibilidade de algum enviesamento dos resultados ^20^ ^,^ ^21^ ^,^ ^22^ ^,^ ^23^.

A análise dos dados foi realizada no software estatístico IBM SPSS, versão 21.0 (https://www.ibm.com/).

### Aspectos éticos

O estudo foi aprovado por Comitê de Ética em Pesquisa (protocolo nº 5.258.142), tendo seguido *Resolução nº 466/2012* do Conselho Nacional de Saúde para pesquisa envolvendo seres humanos.

## Resultados

Participaram do estudo 563 entregadores, que eram predominantemente homens (96,3%), jovens, tendo metade deles menos de 33 anos e quase 80% menos de 40 anos.

Aproximadamente 70% dos entregadores eram pardos (47,3%) ou pretos (23%), e tinham o Ensino Médio completo. Houve participantes de todas as regiões do Brasil, abarcando 21 estados e o Distrito Federal, com predomínio das regiões Nordeste (46,2%) e Sudeste (36,5%) (Tabela 1).

**Tabela 1:** Prevalência de acidente de trabalho de acordo com características sociodemográficas entre entregadores. *EpisSAT Entregadores*, Brasil, 2022 (N = 563).

Variáveis	População total		Prevalência de pelo menos um acidente de trabalho no último ano	
	n	%	n	%
Sexo [n = 560]				
Masculino	542	96,8	238	43,9
Feminino	18	3,2	10	55,6
Idade (anos) [n = 563]				
15 a 28	184	32,7	100	54,6
> 28 a 37	200	35,5	85	41,9
> 37	179	31,8	63	34,8
Raça/Cor da pele [n = 562]				
Preta	129	23,0	67	51,9
Parda	266	47,3	112	42,1
Branca	145	25,8	60	41,4
Amarela	15	2,7	5	33,3
Indígena	7	1,2	3	42,9
Escolaridade [n = 562]				
Ensino Médio completo ou mais	172	30,6	74	43,0
Até Ensino Médio completo	390	69,4	174	44,6
Região do Brasil em que atua [n = 561]				
Centro-oeste	40	7,1	24	60,0
Nordeste	259	46,2	119	45,9
Norte	11	2,0	4	36,4
Sudeste	205	36,5	77	37,6
Sul	46	8,2	23	50,0

Quase todos os entregadores referem utilizar o capacete (88%) durante o trabalho, entretanto, 67,6% admitem ultrapassagem do sinal vermelho no trânsito. Na jornada diária, mais de 83% dos trabalhadores não contam com o apoio da empresas-plataforma de entrega para resolução dos problemas enfrentados no trabalho. Ademais, 72% relataram conflitos com os clientes das empresas e 70,5% vivenciam conflitos com os fornecedores das mercadorias (Tabela 2).

**Tabela 2:** Prevalência de acidente de trabalho de acordo com características ocupacionais, entre entregadores. *EpisSAT Entregadores*, Brasil, 2022 (N = 563).

Variáveis	População total		Prevalência de pelo menos um acidente de trabalho no último ano	
	n	%	n	%
Inserção como entregador [n = 449]				
Plataforma digital	391	87,1	165	42,2
Outras formas de inserção	58	12,9	25	43,1
Principal atividade [n = 559]				
Sim	503	90,0	227	45,1
Não	56	10,0	18	32,1
Única atividade [n = 558]				
Sim	435	78,0	197	45,3
Não	123	22,0	49	39,8
Tempo de trabalho como entregador (anos) [n = 550]				
Até 3	303	53,8	143	47,2
De 4 a 7	120	21,3	59	49,9
Acima de 7	140	24,9	46	32,9
Jornada semanal (dias) [n = 553]				
Até 5	168	30,4	67	39,9
6	149	26,9	60	40,3
7	236	42,7	118	50,0
Jornada diária (horas) [n = 508] *				
Até 8	148	29,1	59	39,9
Acima de 8 até 10	134	26,4	59	44,0
Acima de 10	226	44,5	111	49,1
Veículo utilizado [n = 563] **				
Bicicleta	65	11,6	26	40,0
Motocicleta	495	88,4	222	44,8
Característica do veículo utilizado [n = 561]				
Próprio	531	94,7	233	43,9
Alugado	23	4,1	10	43,5
Outros	07	1,2	4	57,1
Armazenamento das mercadorias [n = 562]				
*Bag*	331	58,9	168	50,8
Baú fixo	212	37,7	77	36,3
Outros	19	3,3	3	15,8
Contribuição para o INSS [n = 556]				
Sim	280	50,4	103	36,8
Não	276	49,6	143	51,8
Utilização do capacete [n = 523]				
Sim	460	88,0	210	45,7
Não	63	12,0	26	41,3
Ultrapassagem de sinal vermelho [n = 552]				
Sim	373	67,6	191	51,2
Não	179	32,4	54	30,2
Apoio da empresa [n = 561]				
Frequentemente	93	16,6	31	33,3
Raramente/Nunca	468	83,4	216	46,2
Apoio dos colegas [n = 556]				
Frequentemente	282	50,6	129	45,7
Raramente/Nunca	275	49,4	117	42,5
Ritmo acelerado [n = 558]				
Frequentemente	389	69,7	190	48,8
Raramente/Nunca	169	30,3	57	33,7
Conflito com fornecedores [n = 556]				
Sim	392	70,5	191	48,7
Não	164	29,5	56	34,1
Conflito com clientes das empresas [n = 550]				
Sim	396	72,0	203	51,3
Não	154	28,0	43	27,9
População total	563	100,0	248	44,1

INSS: Instituto Nacional do Seguro Social.

* Houve 13 casos ausentes e foram excluídos quatro indivíduos que não trabalharam na última semana e 38 com carga horária diária de trabalho superior a 15 horas;

** Foram excluídos três indivíduos que utilizavam outro tipo de veículo.

Os entregadores mais jovens, os que trabalham sete dias por semana, os que realizam ultrapassagem do sinal vermelho e aqueles que relatam conflitos com clientes apresentaram as maiores prevalências de pelo menos um acidente de trabalho, igual ou superiores a 50%, ampliada conforme o aumento da jornada de trabalho diária (Tabelas 1 e 2).

Os entregadores realizam entregas como atividade remunerada principal (90%) ou como única atividade remunerada (78%), inseridos principalmente em empresas-plataforma de entrega (87,1%). Cerca de 70% dos entregadores trabalha seis ou sete dias por semana com jornada diária de dez horas ou mais para 50% dos participantes (Tabela 2). Entre esses, 37,2% trabalharam 12 horas ou mais na última semana (dados não apresentados em tabela). Proporção relevante dos trabalhadores ingressou na categoria há até três anos (53,8%), o que corresponde, principalmente, ao período pandêmico. Quase todos realizam entregas com motocicleta (88,4%). Cerca de metade não contribui para a Previdência Social.

Verificou-se prevalência de 44,1% de pelo menos um acidente de trabalho no último ano com entregadores. Os acidentes de trânsito representaram 82,8% desses acidentes de trabalho. As regiões corporais mais afetadas foram pernas (27,7%), braços (21,1%), mãos e dedos (19,4%) e pés (16,5%). O afastamento do trabalho devido ao acidente de trabalho foi referido por mais da metade dos entregadores acidentados (56,1%), e entre aqueles que se afastaram por 15 dias ou mais (22,2%), apenas 20% receberam benefício previdenciário. Independente do tempo de afastamento, a ajuda de familiares para custeio das despesas foi a mais relatada (38,3%). Os serviços de saúde utilizados em razão do acidente de trabalho foram quase sempre públicos (84,8%), de urgência e emergência (92,6%) (dados não apresentados em tabelas).

Dos resultados da análise múltipla, destacam-se alguns achados pela pronunciada magnitude de associação.

A experiência de conflitos com clientes das empresas-plataforma de entrega se associou a quase o dobro da ocorrência de acidentes de trabalho sem afastamento quando comparado com aqueles que negaram esses conflitos (RP = 1,96). Ter o trabalho de entregas como atividade remunerada principal e não contar com o apoio da empresa para resolução de problemas decorrentes do trabalho aumentou em 60% e em quase 50%, respectivamente, a ocorrência de acidente de trabalho sem afastamento. Outra associação com esse desfecho foi raça/cor da pele preta (RP = 1,42) (Tabela 3).

Ultrapassar o sinal vermelho no trânsito representou aumento de quase 80% na prevalência de acidentes de trabalho com afastamento, ao qual também se associou a variável idade, com entregadores com até 28 anos de idade apresentando prevalência 60% maior do que os de maior idade (Tabela 3).

**Tabela 3:** Fatores associados aos acidentes de trabalho conforme necessidade de afastamento do trabalho no último ano. *EpisSAT Entregadores*, Brasil, 2022 (N = 563).

Variáveis	**Acidente de trabalho sem afastamento *vs.* sem acidente de trabalho** **[n = 423]**					**Acidente de trabalho com afastamento *vs.* sem acidente de trabalho** **[n = 453]**				
	Prevalência (%)	RP bruta	IC95%	RP ajustada	IC95%	Prevalência (%)	RP bruta	IC95%	RP ajustada	IC95%
Idade (anos)										
> 37	20,0	1,00	-	1,00	-	22,1	1,00	-	1,00	-
> 28 a 37	27,2	1,35	0,83-2,21	1,29	0,77-2,19	26,5	1,20	0,74-1,94	1,10	0,65-1,87
15 a 28	30,0	1,50	0,90-2,48	1,14	0,64-2,04	43,1	1,95	1,26-3,03	1,61	0,95-2,73
Raça/Cor da pele										
Não pretos	23,6	1,00	-	1,00	-	28,3	1,00	-	1,00	-
Pretos	32,6	1,38	0,91-2,11	1,42	0,90-2,25	37,4	1,32	0,90-1,92	1,21	0,80-1,84
Principal atividade										
Não	15,6	1,00	-	1,00	-	22,4	1,00	-	1,00	-
Sim	26,6	1,71	0,79-3,68	1,60	0,63-4,06	31,2	1,39	0,75-2,57	1,38	0,69-2,79
Tempo de trabalho como entregador (anos)										
Acima de 7	18,3	1,00	-	1,00	-	20,3	1,00	-	1,00	-
Até 7	28,2	1,55	0,96-2,49	1,36	0,77-2,42	34,0	1,67	1,08-2,60	1,17	0,68-2,02
Jornada semanal (dias)										
Até 6	23,7	1,00	-	1,00	-	26,1	1,00	-	1,00	-
7	29,3	1,24	0,85-1,81	1,05	0,68-1,61	36,6	1,40	1,00-1,96	1,14	0,78-1,67
Jornada diária (horas)										
Até 8	20,5	1,00	-	1,00	-	28,2	1,00	-	1,00	-
Acima de 8 até 10	24,2	1,18	0,67-2,09	1,05	0,57-1,93	31,8	1,13	0,71-1,80	1,15	0,71-1,88
Acima de 10	31,1	1,52	0,93-2,48	1,16	0,67-2,01	33,5	1,19	0,78-1,81	0,92	0,58-1,46
Ultrapassagem do sinal vermelho										
Não	18,3	1,00	-	1,00	-	17,2	1,00	-	1,00	-
Sim	30,3	1,65	1,07-2,54	1,37	0,85-2,21	37,7	2,19	1,43-3,35	1,78	1,11-2,84
Apoio da empresa										
Frequentemente	16,2	1,00	-	1,00	-	23,5	1,00	-	1,00	-
Raramente/Nunca	27,4	1,69	0,93-3,08	1,49	0,78-2,85	32,1	1,37	0,84-2,22	1,28	0,75-2,19
Ritmo acelerado										
Raramente/Nunca	20,0	1,00	-	1,00	-	20,6	1,00	-	1,00	-
Frequentemente	28,7	1,43	0,93-2,20	1,05	0,65-1,71	35,2	1,71	1,13-2,58	1,36	0,84-2,21
Conflitos com clientes										
Não	12,6	1,00	-	1,00	-	19,7	1,00	-	1,00	-
Sim	32,3	2,56	1,51-4,36	1,96	1,12-3,45	36,1	1,84	1,21-2,81	1,29	0,81-2,02

IC95%: intervalo de 95% de confiança; RP: razão de prevalência

Realizar entregas como atividade principal e há até sete anos representou aumento em quase 80% e 50%, respectivamente, nos acidentes de trabalho que não levaram ao uso de serviços de saúde. Ultrapassar o sinal vermelho também se associou à ocorrência do acidente de trabalho que requereu a busca por serviço de saúde (RP = 1,75) ou não (RP = 1,41). Novamente, os entregadores mais jovens se vitimaram mais por acidente de trabalho e em eventos de suposta maior gravidade por determinarem a busca de atendimento (RP = 1,65). Enquanto a experiência de conflitos com clientes das empresas se associou aos acidentes de trabalho que resultaram ou não no uso de serviço de saúde (RP = 1,46 e RP = 1,66, respectivamente), a ausência de apoio da empresa se associou, principalmente, ao acidente de trabalho que levou à busca por atendimento (RP = 1,45) (Tabela 4).

**Tabela 4:** Fatores associados aos acidentes de trabalho conforme o uso de serviço de saúde no último ano. *EpisSAT Entregadores*, Brasil, 2022.

Variáveis	**Acidente de trabalho sem uso de serviço de saúde *vs.* sem acidente de trabalho** **[n = 438]**					**Acidente de trabalho com uso de serviço de saúde *vs.* sem acidente de trabalho** **[n = 440]**				
	Prevalência (%)	RP bruta	IC95%	RP ajustada	IC95%	Prevalência (%)	RP bruta	IC95%	RP ajustada	IC95%
Idade (anos)										
> 37	23,4	1,00	-	1,00	-	20,0	1,00	-	1,00	-
> 28 a 37	25,3	1,12	0,69-1,84	1,03	0,62-1,71	28,6	1,40	0,86-2,27	1,41	0,82-2,42
15 a 28	37,3	1,67	1,06-2,62	1,23	0,73-2,06	37,3	1,86	1,17-2,97	1,65	0,93-2,91
Raça/Cor da pele										
Não pretos	26,2	1,00	-	1,00	-	26,2	1,00	-	1,00	-
Pretos	34,7	1,32	0,89-1,97	1,24	0,79-1,95	35,4	1,35	0,91-2,00	0,74	0,48-1,13
Principal atividade										
Não	17,4	1,00	-	1,00	-	20,8	1,00	-	1,00	-
Sim	29,0	1,67	0,81-3,42	1,79	0,71-4,45	29,2	1,40	0,73-2,68	1,23	0,61-2,50
Tempo de trabalho como entregador (anos)										
Acima de 7	19,0	1,00	-	1,00	-	20,3	1,00	-	1,00	-
Até 7	31,4	1,65	1,04-2,62	1,50	0,85-2,64	31,4	1,54	0,99-2,61	0,99	0,57-1,49
Jornada semanal (dias)										
Até 6	26,6	1,00	-	1,00	-	23,4	1,00	-	1,00	-
7	31,0	1,16	0,81-1,66	0,93	0,62-1,40	35,5	1,52	1,07-2,16	1,33	0,89-1,99
Jornada diária (horas)										
Até 8	23,3	1,00	-	1,00	-	26,4	1,00	-	1,00	-
Acima de 8 até 10	30,6	1,31	0,79-2,18	1,29	0,75-2,22	25,7	0,97	0,58-1,63	0,92	0,54-1,57
Acima de 10	31,5	1,35	0,85-2,15	1,11	0,67-1,86	33,5	1,27	0,82-1,95	0,93	0,58-1,49
Ultrapassagem do sinal vermelho										
Não	18,8	1,00	-	1,00	-	16,7	1,00	-	1,00	-
Sim	33,8	1,80	1,18-2,72	1,41	0,89-2,23	35,0	2,10	1,35-3,26	1,75	1,08 -2,84
Apoio da empresa										
Frequentemente	20,5	1,00	-	1,00	-	19,5	1,00	-	1,00	-
Raramente/Nunca	29,6	1,44	0,85-2,44	1,25	0,70-2,23	30,4	1,56	0,91-2,67	1,45	0,80-2,61
Ritmo acelerado										
Raramente/Nunca	18,8	1,00	-		-	21,7	1,00	-	1,00	-
Frequentemente	32,8	1,74	1,13-2,68	1,28	0,78-2,08	31,8	1,47	0,98-2,21	1,16	0,72-1,86
Conflitos com clientes										
Não	22,9	1,00	-	1,00	-	18,2	1,00	-	1,00	-
Sim	31,2	2,17	1,36-3,48	1,66	0,99-2,76	33,2	2,08	1,31-3,29	1,46	0,90-2,35

IC95%: intervalo de 95% de confiança; RP: razão de prevalência.

A ultrapassagem do sinal vermelho no trânsito e realizar entregas como atividade principal aumentaram a prevalência de um acidente de trabalho no último ano em, aproximadamente, 60%, mas também se associaram à ocorrência de dois ou mais acidentes de trabalho no último ano (RP = 1,53 e RP = 1,41 respectivamente). Sofrer dois ou mais acidentes de trabalho no último ano também ocorreu mais entre aqueles que trabalham sem contar com apoio da empresa (RP = 1,66), com ritmo acelerado (RP = 1,56), e que são mais jovens (RP = 1,44). Novamente, a experiência de conflito com clientes das empresas se associa à ocorrência de múltiplos acidentes de trabalho no período, quase dobrando a prevalência ao se comparar com entregadores que não viveram essa situação estressora (RP = 1,90) (Tabela 5).

**Tabela 5:** Fatores associados a um ou dois ou mais acidentes de trabalho no último ano. *EpisSAT Entregadores*, Brasil, 2022.

Variáveis	**Um acidente de trabalho no último ano *vs.* sem acidente de trabalho** **[n = 419]**					**Dois ou mais acidentes de trabalho *vs.* sem acidente de trabalho** **[n = 458]**				
	Prevalência (%)	RP bruta	IC95%	RP ajustada	IC95%	Prevalência (%)	RP bruta	IC95%	RP ajustada	IC95%
Idade (anos)										
> 37	21,0	1,00	-	1,00	-	22,1	1,00	-	1,00	-
> 28 a 37	25,2	1,26	0,75-2,11	1,19	0,69-2,07	27,5	1,25	0,79-2,00	1,20	0,73-1,98
15 a 28	29,1	1,48	0,89-2,47	1,32	0,72-2,42	44,4	1,92	1,25-2,95	1,44	0,88-2,37
Raça/Cor da pele										
Não pretos	23,8	1,00	-	1,00	-	28,3	1,00	-	1,00	-
Pretos	28,7	1,21	0,77-1,89	1,34	0,82-2,18	40,4	1,43	0,99-2,04	1,27	0,85-1,89
Principal atividade										
Não	15,6	1,00	-	1,00	-	22,4	1,00	-	1,00	-
Sim	26,0	1,67	0,78-3,6	1,58	0,63-3,99	32,0	1,43	0,77-2,64	1,41	0,70-2,85
Tempo de trabalho como entregador (anos)										
Acima de 7	20,3	1,00	-	1,00	-	19,0	1,00	-	1,00	-
Até 7	26,6	1,31	0,83-2,06	1,22	0,69-2,15	35,4	1,86	1,18-2,94	1,28	0,74-2,21
Jornada semanal (dias)										
Até 6	24,9	1,00	-	1,00	-	25,2	1,00	-	1,00	-
7	24,8	1,00	0,7-1,49	0,85	0,54-1,33	39,8	1,58	1,13-2,20	1,33	0,92-1,94
Jornada diária (horas)										
Até 8	19,8	1,00	-	1,00	-	29,4	1,00	-	1,00	-
Acima de 8 até 10	27,9	1,41	0,81-2,45	1,39	0,77-2,50	28,6	0,97	0,6-1,57	0,88	0,53-1,44
Acima de 10	27,2	1,37	0,82-2,29	1,22	0,70-2,12	36,8	1,25	0,84-1,87	0,86	0,55-1,35
Ultrapassagem do sinal vermelho										
Não	17,2	1,00	-	1,00	-	18,3	1,00	-	1,00	-
Sim	30,0	1,74	1,18-2,72	1,61	0,99-2,62	38,1	2,08	1,38-3,15	1,53	0,97-2,41
Apoio da empresa										
Frequentemente	21,5	1,00	-	1,00	-	17,3	1,00	-	1,00	-
Raramente/Nunca	25,4	1,18	0,7-1,99	1,13	0,64-1,98	34,0	1,96	1,11-3,47	1,66	0,90-3,06
Ritmo acelerado										
Raramente/Nunca	22,8	1,00		1,00	-	17,6	1,00	-	1,00	-
Frequentemente	26,3	1,15	0,76-1,75	0,97	0,60-1,56	37,2	2,11	1,36-3,27	1,56	0,94-2,58
Conflitos com clientes										
Não	17,8	1,00	-	1,00	-	14,6	1,00	-	1,00	-
Sim	29,3	1,65	1,04-2,06	1,24	0,76-2,02	38,7	2,65	1,63-4,30	1,90	1,14-3,17

IC95%: intervalo de 95% de confiança; RP: razão de prevalência.

Assim, foram evidenciadas como variáveis que de forma mais consistente se associaram à ocorrência de acidentes de trabalho, a experiência de conflito com clientes das empresas, o trabalho de entregas como atividade remunerada principal e a ultrapassagem de sinal vermelho no trânsito. Ademais, ser mais jovem, de raça/cor da pele preta, trabalhar como entregador há até sete anos, trabalhar em ritmo acelerado e não receber apoio da empresa para solução de problemas decorrentes do trabalho se associaram ao acidente de trabalho, seja com o evento de menor ou de suposta maior gravidade.

## Discussão

O estudo evidenciou elevada prevalência de acidentes de trabalho, chegando a 54,6% entre aqueles com até 28 anos de idade, 50% entre quem trabalha sete dias por semana e 49,1% entre quem trabalha mais de dez horas por dia. Aqueles que vivenciaram conflito com clientes das empresas-plataforma de entrega tiveram prevalência de acidentes de trabalho de 51,3%. Essa elevada magnitude, não identificada em estudos com outras categorias profissionais, evidencia a alta acidentalidade imposta pelo trabalho precário em empresas-plataforma de entrega às quais se vinculam 87,1% dos entregadores deste estudo, e executado nas ruas, haja vista que 82,8% dessas ocorrências foram acidentes de trânsito com recorrência relevante.

Contrariando o discurso das empresas de que a inserção dos entregadores se daria com flexibilidade de horários e de forma complementar a outras ocupações principais, verificou-se que essa se constitui na única ou principal fonte de renda de uma grande e crescente força de trabalho no país ^24^ ^,^ ^25^. A magnitude desse grupo engajado no trabalho de empresas-plataforma de entrega de mercadorias já é uma realidade a exigir a regulamentação das condições de trabalho da categoria ^7^ ^,^ ^9^.

Essa população que labora no espaço das ruas vem se constituindo em um grupo especialmente vulnerabilizado como vítima de acidentes de trânsito. Os dados oficiais, em que pese a sua fragilidade quanto à caracterização da variável ocupação, evidenciam a alta proporção de acidentes de trânsito acometendo motociclistas, o que chega à proporção de 30% para mortalidade, com tendência crescente, particularmente, em países de média e baixa renda ^2^.

Estudar a ocorrência de acidentes de trabalho exige conhecer a jornada de trabalho à qual a população sob estudo está subordinada. Os nossos achados revelam a intensa exposição ocupacional sob condições adversas do trabalho precário nas ruas, resultando em repercussões relevantes na saúde e na segurança dos entregadores ^4^ ^,^ ^26^.

A jornada diária constatada no presente estudo é muito alta, superior às encontradas na literatura com outras categorias. E mesmo em estudo realizado no início da pandemia de COVID-19 com entregadores, evidencia-se uma jornada menos extensa àquela altura ^10^. Desde então, para além da discussão da jornada extenuante dessa categoria, sua tendência crescente tem sido considerada consequência do aumento da concorrência diante da ampliação do contingente de entregadores, em face das crises econômica e sanitária, mas também do rebaixamento da remuneração promovido pelas empresas-plataforma de entrega, ao monopolizarem o setor de entregas e intensificarem a dependência do entregador a esse tipo de serviço ^5^ ^,^ ^10^.

O trabalho no trânsito, conduzindo veículos de duas rodas, é repleto de estressores gerados em disputa das vias públicas com veículos de quatro rodas, sob ameaça constante de um acidente, haja vista a inexistência de vias segregadas para motociclistas ou de um sistema seguro que acomode, sem riscos, os deslocamentos ^2^. A esses estressores se somam aqueles encontrados em serviços de atendimento ao público, quando o entregador está exposto às manifestações de todo o tipo de insatisfação de um amplo e diversificado contingente de clientes das empresas ^27^. Nesse contexto desfavorável do trabalho nas ruas e no trânsito, destaca-se ainda a escassez de apoio da empresas-plataforma de entrega para solução dos problemas enfrentados no trabalho, ao lidarem com adversidades relativas à plataforma digital e, principalmente, na relação com clientes.

A prevalência de acidentes de trabalho encontrada no presente estudo foi mais de dez vezes aquela da população geral ocupada no Brasil ^28^ e superior à verificada em ambientes fechados, a exemplo de trabalhadores de saúde, cuja frequência de acidentes de trabalho com material biológico no último ano correspondeu a 3,4% ^16^. Entre mototaxistas, a frequência de acidentes de trabalho variou de 10,5% ^29^ a 26,8% ^30^. Estudo conduzido com entregadores motociclistas por Silva et al. ^31^, anteriormente à expansão das empresas-plataforma de entrega no Brasil, verificou frequência de pelo menos um acidente de trabalho no trânsito, particularmente, de 37,1%. A magnitude desses achados, que já é elevada, ainda é menor que a do presente estudo, mas reforça a condição insegura de trabalho no trânsito sob motocicletas, principalmente.

Quanto à gravidade do acidente de trabalho, mais da metade dos entregadores acidentados se afastou do trabalho. Entre aqueles com 15 dias ou mais de afastamento, 80% não tiveram o direito a um período remunerado pela Previdência Social para recuperação da incapacidade gerada pelo acidente de trabalho, em situação de desproteção social, na qual a sobrevivência se torna um desafio para esses trabalhadores. Ademais, a busca por serviços de saúde majoritariamente públicos, de urgência e emergência, dada a natureza aguda do evento, indica a ampliação da demanda e dos custos para o Sistema Único de Saúde (SUS) frente às condições do trabalho plataformizado e seu impacto na saúde.

A diversidade na abordagem da gravidade dos acidentes de trabalho na literatura limita as possibilidades de comparação ^31^ ^,^ ^32^. É provável que a gravidade dos acidentes de trabalho, estimada no presente estudo por meio da referência ao afastamento do trabalho e ou à utilização de serviço de saúde, esteja subestimada, considerando a desproteção social e a provável menor acessibilidade aos serviços de saúde entre trabalhadores plataformizados ^33^. A ausência ou dificuldade de acesso ao seguro indenizatório proveniente das empresas-plataforma de entrega, e o acesso escasso à Previdência Social, podem fazer com que entregadores evitem o afastamento ou o façam por um período menor do que o recomendado para recuperação da saúde, em virtude da necessidade de auferir renda para sobreviver ^5^ ^,^ ^31^ ^,^ ^32^. A busca por serviços de saúde também pode ser desestimulada frente ao imperativo de seguir trabalhando ^5^ ^,^ ^33^. Dados da *Pesquisa Nacional de Saúde* ^34^ permitiram estimar que o acesso precário aos serviços de saúde é maior entre homens, pessoas com idade de 18 a 24 anos, negras, e de baixa posição socioeconômica, perfil semelhante ao dos entregadores deste estudo.

A ultrapassagem do sinal vermelho no trânsito é referida em outros estudos com entregadores, em acordo com nossos achados, relacionando-se com a pressão de tempo imposta pelas empresas-plataforma de entrega e que se torna mais efetiva por meio das tecnologias das plataformas digitais, com remuneração por entrega finalizada e um sistema de avaliação que condiciona o recebimento de novos pedidos de entrega, entre outros fatores, à agilidade do serviço prestado ^5^ ^,^ ^26^ ^,^ ^32^ ^,^ ^33^. No entanto, a adesão ao uso do capacete, um equipamento de proteção individual, por 88% dos entregadores no presente estudo evidencia que as condições objetivas de trabalho é que podem favorecer ou dificultar a adoção de medidas de segurança pelos trabalhadores, ou seja, nas condições reais estão os determinantes de um sistema de trabalho mais ou menos seguro ^32^ ^,^ ^35^.

Sob pressão de tempo, ultrapassar o sinal pode ser a única alternativa, enquanto o uso do capacete, que muitas vezes é mantido em posição na cabeça, mesmo ao sair da motocicleta para receber ou entregar a mercadoria, encontra factibilidade mesmo no trabalho intensificado.

Ser mais jovem foi associado aos acidentes de trabalho com afastamento, com uso de serviço de saúde e recorrentes no período de um ano. A associação entre trabalhadores mais jovens e acidentes de trabalho está em acordo com estudos prévios com entregadores ^31^ ^,^ ^32^ ^,^ ^33^. Esses achados não parecem estar vinculados à juventude em si, mas a menores oportunidades de capacitação em trabalho precário ^33^ ^,^ ^35^ ^,^ ^36^.

Nesse sentido, a maior susceptibilidade para intensificar o trabalho, adotar mais altas velocidades, ter menor atenção às regras de trânsito em razão da ausência de capacitação para atividade profissional com motocicleta e o menor tempo de experiência no trabalho são algumas explicações para o maior acometimento nesse segmento etário ^3^ ^,^ ^33^ ^,^ ^35^. Na Grécia, entregadores com 18 a 24 anos apresentaram mais que o dobro do risco (120% a mais) de ultrapassar o sinal vermelho ^32^. Em outro inquérito, com 1.310 entregadores com benefícios securitários concedidos após acidente motociclístico na Coreia, verificou-se que ainda que trabalhadores com menos de 20 anos apresentassem prevalência relevante de acidentes de trabalho, esta foi mais baixa que aquela entre trabalhadores com mais de 30 anos, o que sugere a subnotificação dos eventos entre os entregadores mais jovens, com menos acesso aos benefícios securitários e, portanto, sub-representados na população estudada ^36^.

No presente estudo, não houve diferença na frequência de acidentes de trabalho entre brancos e pardos, motivo pelo qual foram analisados, de forma combinada, como não pretos, assim como os amarelos e os indígenas, ainda que se reconheçam as desvantagens entre pretos e pardos no mundo do trabalho. Os entregadores de raça/cor da pele preta foram mais vitimados pelo acidente de trabalho que cursou sem afastamento.

Realizar entregas como atividade remunerada principal se associou, principalmente, ao acidente de trabalho de suposta menor gravidade e que não foi recorrente no período de um ano. A dependência dessa fonte de renda pode potencializar a intensificação do trabalho, culminando em extensas jornadas de trabalho que tem sido associada aos acidentes de trabalho ^5^. A potencialização dos efeitos dos estressores do trabalho em jornadas extenuantes e a fadiga decorrente da manutenção de posturas anômalas favorecem condições inseguras ao pilotar ^26^ ^,^ ^33^ ^,^ ^37^ ^,^ ^38^.

Realizar entregas há até sete anos se associou ao acidente de trabalho sem uso de serviço de saúde. Esse período coincide com a expansão das empresas-plataforma de entrega, sugerindo que não apenas o menor tempo de experiência, mas também a inserção na categoria nesse contexto de precarização ampliada pode ter comprometido a segurança no trabalho sob veículo de duas rodas ^5^ ^,^ ^7^ ^,^ ^36^ ^,^ ^37^.

Os estressores psicossociais no trabalho foram associados aos acidentes de trabalho, incluindo suas repercussões de maior gravidade. Quando presente, o apoio social possibilita, em alguma medida, proteger o trabalhador dos efeitos da elevada carga psicológica em condição de baixa autonomia para gestão do próprio trabalho, como ocorre na atividade de entregadores ^39^. Entre trabalhadores da saúde, a experiência de baixo apoio contribuiu para o aumento em 24% da ocorrência de acidentes de trabalho com material biológico, comparada com o alto apoio social ^16^. Associou-se, ainda, com a maior ocorrência de acidentes de trabalho no trânsito em dois de três grupos de motoristas profissionais - de ônibus urbano e interurbano ^40^.

O ritmo acelerado de trabalho, que se associou à recorrência do acidente de trabalho, é consequência da exigência de tempo no trabalho. A avaliação pelos clientes das empresas, um dos componentes de um escore que condiciona a oferta de serviço, inclui pontualidade na entrega a partir de tempos de deslocamentos estimados pelas empresas ^33^ ^,^ ^41^. Ademais, a elevada produtividade exigida, com aplicação de metas gamificadas, estimula a aceleração do ritmo de trabalho ^41^. Esse cenário favorece o emprego de velocidades elevadas em veículo motorizado, reconhecido fator associado aos acidentes de trânsito e à adoção de comportamentos relacionados à maior gravidade do acidente de trabalho ^26^ ^,^ ^32^. Chen ^37^ constatou que a pressão de tempo no trabalho, além de ser o maior preditor isolado do estresse, potencializou a influência da sobrecarga sobre esse agravo à saúde. Por conseguinte, isso contribuiu para desenvolvimento de comportamento considerado inseguro durante a condução no trânsito, como a ultrapassagem do sinal vermelho.

No presente estudo, a ultrapassagem do sinal vermelho foi associada aos acidentes de trabalho com maior gravidade. Conhecer as condições reais nas quais entregadores realizam suas tarefas é necessário a fim de evitar reduzir a complexidade dos achados com explicações da esfera individual e comportamental, sem identificação dos determinantes econômicos e sociais do trabalho intensificado ^32^.

A rua é um ambiente de trabalho inóspito, no qual os trabalhadores estão isolados e sem os recursos usualmente encontrados em ambientes de trabalho delimitados, como a fábrica ou o escritório, para lidar com situações conflituosas ^6^. Tendo em vista a associação verificada entre os estressores psicossociais e os diferentes desfechos relacionados ao acidente de trabalho, especialmente os conflitos com clientes, mecanismos para assegurar o apoio aos entregadores para resolução de problemas referentes ao trabalho, considerando o espaço da rua, devem ter algum efeito protetor. Esse apoio pode contribuir para a redução dos conflitos com clientes.

Houve uma concentração de acidentes de trabalho no trânsito. Outros estudos epidemiológicos devem investigar as diferentes modalidades de acidentes de trabalho e a reconhecida subnotificação da violência interpessoal enquanto acidente de trabalho no espaço da rua ^6^. Ademais, é possível que o medo de sofrer uma violência contribua com a ultrapassagem do sinal vermelho no trânsito, associada ao acidente de trabalho neste estudo.

Nossos resultados devem contribuir para a vigilância em saúde do trabalhador, em alinhamento com a agenda global para o desenvolvimento sustentável, que inclui entre seus objetivos o trabalho decente e a ampliação da segurança viária e redução de mortes no trânsito ^12^ ^,^ ^42^.

### Pontos fortes e limitações do estudo

Não foram identificados estudos prévios que tenham investigado a magnitude e os fatores associados aos acidentes de trabalho e às suas repercussões, como afastamento do trabalho e uso de serviços de saúde, no Brasil. Portanto, nossas evidências apresentam uma contribuição inédita à literatura para caracterização e elucidação da magnitude dos acidentes de trabalho com entregadores, especialmente no trabalho plataformizado.

As estratégias adotadas para viabilizar o estudo são um ponto forte, tendo em vista as dificuldades de acesso a essa população e a ausência de disponibilidade das empresas-plataforma de entrega em fornecer à sociedade dados sobre essa força de trabalho ^10^ ^,^ ^15^.

Os procedimentos metodológicos adotados visaram minimizar potenciais vieses de informação e de seleção. É possível que entregadores afastados do trabalho devido ao acidente de trabalho não tenham acessado o *link* para o questionário veiculado em grupos de entregadores no WhatsApp e nas redes sociais. No entanto, a permanência e a recorrência do encaminhamento desse *link* nas mídias sociais da categoria por quatro meses podem ter minimizado esse possível viés de seleção, o efeito sobrevivência do trabalhador sadio.

Diante da impossibilidade de realizar um censo e da estratégia possível de seleção da amostra não probabilística, não é possível se afastar a possibilidade de haver diferenças entre os entregadores participantes e os não participantes, exigindo parcimônia na generalização dos resultados. Mas a não participação não parece ter decorrido de recusa associada a um motivo especial. Ao contrário, é o trabalho precário que desestimula a dedicação de tempo para responder à pesquisa. Nesse sentido, é provável que aqueles que tiveram a oportunidade de ouvir mais sobre o projeto, nas ações presenciais e *online* de divulgação, tenham se sentido mais estimulados a participar. A restrição da divulgação presencial a dois estados decorreu da ausência de financiamento da pesquisa por agências de fomento. Nesse sentido, os resultados alcançados que evidenciam a gravidade da situação de saúde desses trabalhadores compensam o grande esforço empreendido para viabilizar a pesquisa.
